# Associative Mechanisms Allow for Social Learning and Cultural Transmission of String Pulling in an Insect

**DOI:** 10.1371/journal.pbio.1002564

**Published:** 2016-10-04

**Authors:** Sylvain Alem, Clint J. Perry, Xingfu Zhu, Olli J. Loukola, Thomas Ingraham, Eirik Søvik, Lars Chittka

**Affiliations:** 1 Department of Biological and Experimental Psychology, School of Biological and Chemical Sciences, Queen Mary University of London, London, United Kingdom; 2 Xishuangbanna Tropical Botanical Garden, Chinese Academy of Sciences, Menglun Town, Yunnan, P. R. China; 3 F1000Research, London, United Kingdom; 4 Department of Science and Mathematics, Volda University College, Volda, Norway; Centre for Genomic Regulation, SPAIN

## Abstract

Social insects make elaborate use of simple mechanisms to achieve seemingly complex behavior and may thus provide a unique resource to discover the basic cognitive elements required for culture, i.e., group-specific behaviors that spread from “innovators” to others in the group via social learning. We first explored whether bumblebees can learn a nonnatural object manipulation task by using string pulling to access a reward that was presented out of reach. Only a small minority “innovated” and solved the task spontaneously, but most bees were able to learn to pull a string when trained in a stepwise manner. In addition, naïve bees learnt the task by observing a trained demonstrator from a distance. Learning the behavior relied on a combination of simple associative mechanisms and trial-and-error learning and did not require “insight”: naïve bees failed a “coiled-string experiment,” in which they did not receive instant visual feedback of the target moving closer when tugging on the string. In cultural diffusion experiments, the skill spread rapidly from a single knowledgeable individual to the majority of a colony’s foragers. We observed that there were several sequential sets (“generations”) of learners, so that previously naïve observers could first acquire the technique by interacting with skilled individuals and, subsequently, themselves become demonstrators for the next “generation” of learners, so that the longevity of the skill in the population could outlast the lives of informed foragers. This suggests that, so long as animals have a basic toolkit of associative and motor learning processes, the key ingredients for the cultural spread of unusual skills are already in place and do not require sophisticated cognition.

## Abstract 2

Bumblebees can be trained to pull strings to obtain a reward, can learn to string pull through observation, and can culturally spread string pulling throughout a colony.

## Author Summary

Social insects make use of simple mechanisms to achieve many seemingly complex behaviors and thus may be able to provide a unique resource for uncovering the basic cognitive elements required for culture. Here, we first show that bumblebees can be trained to pull a string to access a reward, but most could not learn on their own. Naïve bees learned how to pull strings by observing trained demonstrators from a distance. Learning the behavior through observation relied on bees paying attention to both the string and the position of the trained demonstrator bee while pulling the string. We then tested whether bees could pass this information to others during a semi-natural situation involving several colonies. We found that once one bee knew how to string pull, over time, most of the foraging bees learned from the initially trained bee or from bees who had learned from the trained bee, even after the initial demonstrator was no longer available. These results suggest that learning a nonnatural task in bumblebees can spread culturally through populations.

## Introduction

Social learning is widespread in animals [1,2] and can enable novel behavior routines, sometimes introduced by a single “innovator,” to spread among individuals in a group [1–5]. Examples are potato washing and termite fishing in primates, pinecone stripping in rodents and milk bottle opening in birds [6–10]. Such phenomena in animals have received considerable attention because researchers hoped to discover the key evolutionary ingredients of the cultural processes that define us as humans [11–13]. Social learning can lead to group-specific behavior patterns that are shared by a large number of animals in an area [13–15]. Two key components of culture-like phenomena in animals include spreading of a new behavior via social learning as well as persistence of the behavior in groups for extended periods of time [5,12,14,16,17]. Individuals that picked up the information from the initial demonstrator(s) can themselves become demonstrators to uninformed individuals [18], a process by which such group-specific phenomena can, at least in principle, persist across many generations [5,19].

In some cases, culture-like phenomena such as beach hunting in killer whales (*Orcinus orca*) [17] and lexigram communication in bonobos (*Pan paniscus*) [20] require relatively sophisticated learning mechanisms, for example imitation and/or teaching [21–23]. In insects, seemingly complex processes of social information acquisition, for example the gradual consensus building that occurs when honeybee swarms decide on new nesting locations [24], can sometimes instead be mediated by relatively simple learning mechanisms [25,26], suggesting that cultural processes may not necessarily require sophisticated learning abilities [27–29]. The spread of novel foraging techniques by means of a formal transmission chain experiment [5], in which an experimentally induced innovation is seeded into a group and the subsequent spread is monitored in a social network analysis, has never been explored in an insect. We suggest that doing so provides a unique opportunity for the exploration of the basic cognitive elements required for culture [18,30].

A variety of impressive cognitive skills in social bees has been described, such as object categorization, simple spatial concepts, and numerosity, as well as social learning skills by which bees can acquire information about valuable food sources by observing conspecifics [25,26,31–33]. Scholars in comparative cognition have advocated testing animal intelligence by exploring the flexibility and innovative skills in solving tasks that are relatively remote from the animal’s natural behavior [34,35]. Here, we explore whether bumblebees (*Bombus terrestris*) have the capacity to learn a string pulling task [36]. We also test whether naïve observers can acquire this technique through observation of trained demonstrators, as occurs in naturally widespread foraging techniques [37,38]. Finally, we explore whether and how swiftly such an experimentally introduced innovation can spread through a bumblebee colony. To do this, we used an “open group diffusion” paradigm (forager pairings were not determined by the experimenter) [39] to determine whether diffusion (or transmission) chains that begin with a trained bee follow a sequence in which observers successively become models for a subsequent observer in the chain [5].

## Results

### The Acquisition of a String Pulling Technique by Individual Bumblebees

**Fig 1:**
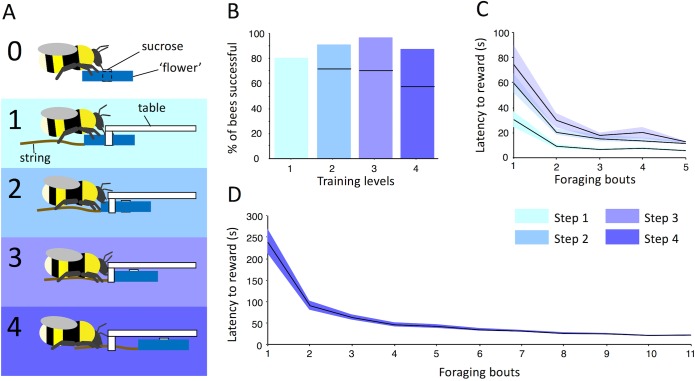
Training bees to pull a string to obtain a reward. (A) Stepwise string pulling training protocol. Successive steps: Step 0, pretraining on blue artificial flowers (note that all bees were trained on this step); Step 1, 50% of the flower covered by the transparent table; Step 2, 75% of the flower covered; Steps 3 and 4, 100% of the flower covered. The flower was positioned at the edge in Step 3 and 2 cm under the table in Step 4. (B) Percentage of successful bees in Steps 1 to 4 (*n* = 40, 32, 29, and 28, respectively). Black horizontal lines within bars indicate the percentage of bees of the original 40. (C) and (D), mean ± standard error (s.e.) (line and shaded area, s) latency to obtain the reward in Steps 1–3 and 4. (C) Mean latency for the five foraging bouts of Steps 1–3. Data points, from left to right, in (D) indicate the latency to reward in Step 4 for the bout with first occurrence of string pulling and the ten foraging bouts that followed. Bees needed 6.17 ± 1.2 foraging bouts before displaying string pulling in Step 4 (see S1 Data).

**Fig 2:**
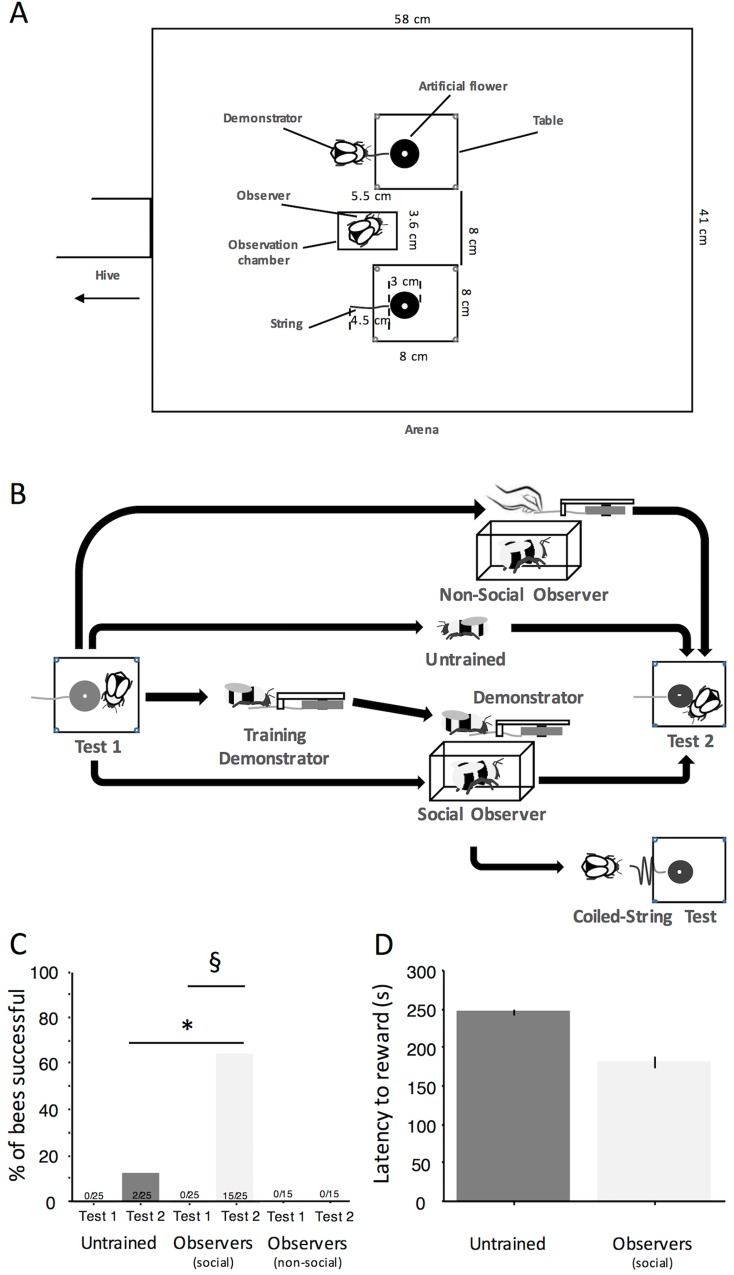
Social transmission of string pulling. (A) Arena set up for the observation of string pulling. (B) The various testing procedures. Tests 1 and 2 were identical and consisted of giving 5 min to individual bees to solve the string pulling task. After having been trained to forage from blue artificial flowers, bees were tested a first time (Test 1). Then, demonstrators were trained (see Fig 1) and used to display string pulling (two instances, straight strings) during each of five foraging bouts to individual observers (*n* = 52) placed in a transparent Plexiglas cage. After the observation phase, 25 observers were tested again with the straight-string task (Test 2) and 27 with the coiled-string task. Fifteen different bees observed the flower moving without visible actor so that a forager could then obtain the sucrose solution (“Ghost control”) and, where tested, with the straight-string task subsequently. Untrained bees (*n* = 25) were also tested a second time with string pulling. (C) Percentage of successful untrained, social, and nonsocial observer bees in Tests 1 and 2. Asterisk: Fisher’s exact test, *p* ≤ 0.0001. Double S: McNemar test, χ^2^_1_ = 13.067, *p* < 0.001. (D) Mean ± s.e. (s) latency in accessing the reward in untrained and observer bees. Observers’ latency was not different from that of the two “innovators” (Mann–Whitney *U* test, U_15_ = 6, *p* = 0.205), (see S1 Data).

To test bees’ capacity to learn the technique of string pulling, we first challenged untrained individuals with a stepwise training procedure (Materials and Methods; S1–S4 Videos). We presented individual bees with three blue artificial flowers with a string attached to each flower and placed under a small transparent Plexiglas table (Materials and Methods). After learning to associate the reward with artificial flowers in a flight arena (Step 0, Fig 1A), but prior to string pulling training, none of the bees from the eight colonies in which individuals were tested singly (*n* = 291) could solve the string pulling task on their first 5-min attempt (Test 1, Fig 2B). Naïve to the string task but attracted to the artificial flowers, these bees tried to reach the reward from the top of the table through the Plexiglas.

In comparison, we were able to train 23 of 40 individuals (Colony 1) through a stepwise training procedure to successfully pull a string to obtain reward (Fig 1B horizontal black bar in column 4, S1–S4 Videos). The stepwise training consisted of four steps of incremental difficulty within which flowers with strings were placed at progressively more distant positions under the transparent table (Steps 1–4, Fig 1A and 1B). On average, successful training for an individual bee took 309 ± 18 min. Gaining access to the reward in the final step required grasping the string with the forelegs and/or mandibles and pulling it closer (S4 Video). The mean time required (latency) to obtain sucrose decreased significantly as a function of experience within each of the four successive training phases (Friedman test, Step 1: *χ*^*2*^_*4*_ = 59.1, *p =* <0.001; Step 2: *χ*^*2*^_*4*_ = 53.1, *p =* <0.001; Step 3: *χ*^*2*^_*4*_ = 52.1, *p =* <0.001; Step 4: *χ*^*2*^_*10*_ = 92.3, *p* < 0.001; Fig 1C and 1D). Eight, three, one, and five individuals gave up at Steps 1, 2, 3, and 4, respectively, either because they ceased foraging activity or had irregular foraging activity (*n* = 11), or because they failed to obtain the reward (*n* = 6). Three of these successfully trained bees were later used as demonstrators in the social learning experiment.

The success of bees learning such a behavior raises the question about the mechanisms by which the demonstrators learned to pull the string. One possibility is that demonstrators are stimulated to repeat the specific sequence of actions (moving the string with their legs) that induces the conditioned stimulus (i.e., the blue flower positioned under the table) to move a little closer. If so, we would expect bees not to move the string with their legs and fail at the task if the colored target stimulus is not present. To test this prediction, we challenged bees (Colony 2) to access the reward when a string was attached to only a colorless inverted Eppendorf cap containing sucrose solution (Materials and Methods) immediately after their initial stepwise training and then again after extensive experience with blue flowers and strings. Without a colored stimulus, only 2 of 15 bees tested obtained the reward after their initial training. We thus hypothesized that relatively inexperienced bees rely on visual feedback of the colored target moving closer while the string is being pulled. To explore this further, we examined the video material for the unsuccessful bees to see if they would attempt to pull the strings and then abort this action when visual feedback was not forthcoming. However, none of the unsuccessful bees demonstrated even an aborted pulling action on the colorless flower’s string. This suggests that most relatively inexperienced bees require the presence of the blue flower to even begin attempting to string pull. (However, there is also evidence for the importance of visual feedback during pulling from an experiment with coiled strings; see section The Mechanisms of Observational Learning in String Pulling.)

Conversely, after 48 h of extensive training (20 instances of string pulling), 11 of the 15 foragers solved the task without feedback from the moving blue flower (S5 Video). Latency to obtaining the reward (147 ± 23.44 s) was much higher than for normal blue flower training (22.1 ± 1.5 s; *t* test: t25 = 6.25, *p* < 0.0001). The subjects’ success differs significantly from their performance when they were relatively inexperienced (McNemar Test, *χ*^*2*^_*1*_ = 7.111, *p* = 0.008), thus indicating that the majority of highly experienced individuals may no longer require visual feedback to perform the necessary sequence of motor actions. In fact, experienced bees may not need the blue flower at all and perhaps have associated the string with the reward.

### Innovators: Rare Individuals that Solved the String Pulling Tasks without Stepwise Training or Social Learning

We gave 50 individuals (Colony 1) the opportunity to solve the string pulling task spontaneously after having learnt that blue flowers are rewarding when they were openly accessible during pretraining (for a 5-min observation period). None of these individuals solved the task. When given a second 5-min opportunity, two of 25 untrained bees succeeded in obtaining the reward (S6 Video). However, they were more than ten times slower at obtaining the reward than experienced string pullers (22.1 ± 1.5 s, mean ± standard error [s.e.], Mann–Whitney *U* test, *U*_*23*_ < 0.001, *p =* 0.024), requiring a relatively long latency of 245 ± 3.53 s. These two bees were exceptionally explorative, trying a wide variety of methods, and solved the task in several attempts by moving the string accidently while trying to reach the flower under the table (see S6 Video and legend for more information). This shows clearly that string pulling can be learned individually by some bumblebees, but this may be an exceptionally rare ability. Across experiments (see below), 291 naïve individuals were tested once, and a total 110 were tested twice, but no further “innovators” were found. In one experiment (the transmission chain experiment below, in which control colonies were not seeded with a skilled demonstrator), bees were given extensive opportunities. After 5 d of foraging, with a maximum number of 18 foraging bouts per individual, no single bee learned to pull the string. Of the 165 bees tested in this experiment in total, nine individuals were tested more than 10 times, and 26 more than 5 times, but all were invariably unsuccessful. Thus, solving a string pulling task spontaneously is a relatively rare occurrence in bumblebees and might either reflect an unusually explorative “personality” in these individuals or simple “luck” in the process of random exploration.

### Social Learning of String Pulling by Caged Observers

We explored whether uninformed bees (Colony 1) could learn this novel foraging technique via observation. After pretraining on blue flowers and Test 1 (Materials and Methods), an uninformed observer bee was placed in a transparent chamber (Fig 2A) where it could observe a demonstrator solve the string pulling task ten times. These observers (*n* = 25) were subsequently tested on the string pulling task alone (Test 2, Fig 2B). In this experiment, observers never interacted directly with demonstrators in the flight arena and had access only to visual social information (S7 Video). Sixty percent of the individuals (15 of 25) that had the opportunity to observe a skilled demonstrator managed to pull the string and obtained the reward on the first trial after having observed the demonstration (Test 2, Fig 2C, S6 Video). These bees, however, were initially almost as slow as the two individuals that solved the tasks without demonstration (181 ± 19 s; Fig 2D). We speculate that the observers picked up the correct location to access the reward from observing skilled demonstrators but did not learn from them the actual technique of string pulling (further explored in the section beneath about the mechanisms of social learning).

We also wished to disentangle the effects of demonstrator copying and object movement copying in how string pulling was learnt by observation. To this end, we used an experimental “ghost control” ([40], S8 Video). We trained 15 nonsocial observers (Colony 3) in exactly the same manner as above with the modification that the flowers were moved without a visible actor: an experimenter pulled the flowers with thin nylon threads attached to the strings while the observers were locked inside the observation chamber (Materials and Methods). Once the string had been pulled, an untrained forager was released into the arena to feed from the now accessible flower. Without direct demonstration of string pulling by a bumblebee forager, none of the observers managed to solve the string pulling task. Nonsocial observers mostly tried to obtain the reward from the top of the table, indicating that the bees need to observe string pulling actions demonstrated by conspecifics to learn the technique. However, because no video material is available to show that observer bees directed their gaze towards the moving flower, it is also possible that in the absence of a conspecific demonstrator, observers simply failed to attend to the movement of the flower.

Finally, because smaller bees might be able to reach further under the table than larger bees, we examined whether body size influenced success in solving the task (Colony 1). Thorax width (as a proxy for body size) was not different between demonstrators (*n* = 40), observers (*n* = 25), and untrained bees (*n* = 25) (ANOVA, *F*_*69*_ = 0.728, *p =* 0.486). Thorax width affected neither demonstrators’ (Student’s *t* test, *t*_*26*_ = 0.659, *p =* 0.516) nor observers’ success rate (Mann–Whitney *U* test, *U*_*23*_ = 79, *p =* 0.846). Similarly, the latency to obtain the reward was not affected by thorax width of demonstrators (Pearson correlation, *r*_*23*_ = -0.086, *p =* 0.696) or observers (Pearson correlation, *r*_*15*_ = 0.375, *p =* 0.169).

### The Mechanisms of Observational Learning in String Pulling

What mechanisms were the observers using to copy the behavior? To answer this question, we explored several associative mechanisms: local enhancement [30,41,42], whereby observers are attracted to the location of their conspecific; stimulus enhancement [30,43], an attraction to the item handled by the demonstrator; and perceptual feedback [44,45], a form of trial-and-error learning in which action causing movement of the rewarding object towards the animal produces positive feedback for continuing that action. We found that all three associative mechanisms were involved in the learning of the string pulling process.

**Fig 3:**
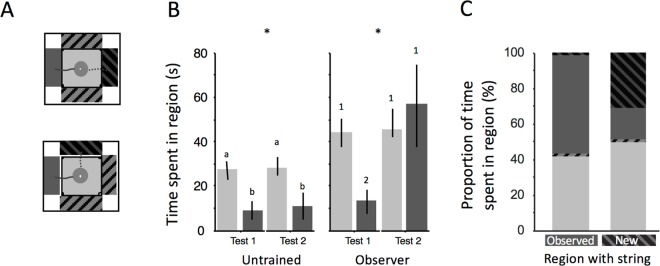
Areas explored by untrained bees and observers of successful string pullers. (A) Regions of interest used for the video analysis of bee behaviors (not true to scale): the original region (where the demonstrator pulled a string, solid dark grey), top region (on the table, solid light grey), the two regions where the string could be presented when it was at variance with the location during the observation phase in the stimulus enhancement tests (thin grey stripes on black) and the adjacent regions where no string was presented (thin black stripes on grey). When testing stimulus enhancement, bees were challenged with a string protruding on the opposite side of one of Plexiglas tables or at 90° compared to the location where it was seen during observational conditioning (dotted lines). Regions were all 16 cm^2^ (adjacent areas: 8 x 2 cm; top region 4 x 4 cm). (B) Mean ± s.e. (s) time spent by unsuccessful observer (*n* = 10) and unsuccessful untrained bees in two of the four regions of interest in their first attempt to retrieve the reward (Test 1) the second attempt (Test 2). Light grey: top of table; dark grey: region where string protruded during observation. Asterisk: Friedman test, *p* < 0.01; letters and figures: post-hoc Tukey test. (C) Percentage of time spent by observer bees in the four regions of interest when the string was protruding in the region where bees had observed demonstrators (left bar, unsuccessful observers, *n* = 10) or the region of the table where the string protruded when it was incongruent with that seen from the observation chamber (right bar, bees tested for stimulus enhancement, *n* = 14). The shades in the various regions of the stacked bars correspond to the shades in Fig 3A (see S1 Data).

To examine the local and stimulus enhancement possibilities, we analyzed the video footage to determine the time bees spent in four different regions of the arena (see Fig 3A, Materials and Methods). In Test 2, unsuccessful observers (*n* = 10, Colony 1) spent more time in the region where the demonstrator was observed (Friedman test, *χ*^*2*^_*3*_ = 14.160, *p =* 0.003, Fig 3B), and untrained bees (*n* = 23, Colony 1) spent more time on top of the table closest to the flower (Friedman test, *χ*^*2*^_*3*_ = 35.162, *p* < 0.001, Fig 3B) than in Test 1, indicating that local enhancement played a part in learning. None of the bees managed to obtain the reward when the string protruded in an area incongruent with that seen during demonstration. However, the string itself also played a role. If the string protruded from a different side of the table compared to the location during the observation period, observer bees (Test 2, *n* = 14, Colony 4; S9 Video) spent more time exploring the region with the string than the region where the demonstrator had been observed (Mann–Whitney *U* test, *U*_*22*_ = 105, *p =* 0.038, Fig 3C), indicating that observers had noticed the string during the observation period and were thus attracted to it. In theory, however, these longer dwelling times in the string region might be explained by bees randomly exploring the edges of the table and simply stopping at a region that contains any protruding object. To explore this possibility, we also evaluated bees’ first approach flights after being released from the observation chamber before they had a chance of interacting with the string. If the string was in the same location as during observation, 92% of observers flew straight to the side of the string. When the location of the string was incongruent with demonstrator location, only 28.5% of observers first visited the region where the demonstrator had been observed (where chance expectation is 25%). The choice frequencies for the four sides of the table are significantly different depending on whether the string was in the correct location (Chi-square of fit, *χ*^*2*^_4_ = 206.857, *p* < 0.0001), indicating that bees were able to see the string from the observation chamber and responded differently when it was presented in an unexpected location. However, there was no appreciable attraction to the string when its location was at variance with that seen from the observation chamber (28.5%). Taken together, these results indicate a strong role for local enhancement (bees were attracted to the location where they had observed a demonstrator) and a subordinate role for stimulus enhancement (bees were attracted to the string when its location was concordant with that during prior observation) [25,46].

Finally, trial-and-error learning was also evident in the learning process. Because individuals might only learn where to obtain the reward and then learn the string pulling by trial-and-error, observer bees (*n* = 27, Colony 5) were tested with a coiled-string paradigm where trial-and-error learning of actions causing the rewarding object moving closer is ineffective. After a standard demonstration of string pulling (Materials and Methods), a 14 cm string was attached to the flower and coiled under the table so that initial tugs on the string would provide no visual feedback of the flower moving closer to the bee. Such coiled-string tests have in the past been used to test whether animals can solve a string pulling puzzle by means-end comprehension, without the perceptual feedback of the reward coming closer [44,45]. Long-tailed macaques (*Macaca fascicularis*) [47] and wolves (*Canis lupus*) [48] have indeed been shown to solve the task even if the string is coiled. However, none of these observer bees were able to solve this task (*n* = 27, Fig 2B, S10 Video), indicating that observers did not glean information about the string pulling technique itself by observing a demonstrator but instead were merely guided to the demonstrator’s previous location (by local enhancement) and the position of the string (stimulus enhancement). The actual act of string pulling relied on individual trial-and-error learning, which in turn necessitates the sensory feedback of tugging on the string, resulting in the target moving closer. We also tested eight experienced individuals (with an experience of more than 20 instances of string pulling) with the coiled-string test; three of these bees succeeded in pulling the coiled string to obtain the reward (S11 Video), indicating that highly experienced individuals do not necessarily require the feedback from seeing the flower move closer while they pull the string. In summary, these results suggest that observational learning of the string pulling task does not involve the “understanding” of the task (“insight”) but the combined use of several simple associative mechanisms and trial-and-error learning.

### The Spread of String Pulling in a Transmission Chain Experiment

**Fig 4:**
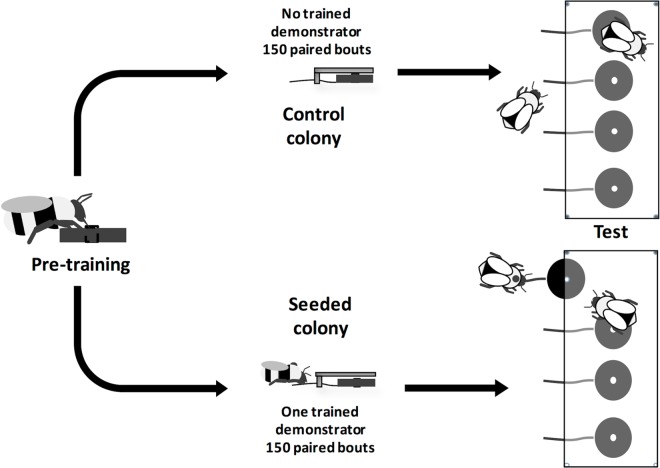
Cultural diffusion paradigm. Bees were group-trained to feed from blue flowers in the foraging arena. Three bees were trained to pull a string to obtain an artificial flower from under a table where they would get reward (sucrose solution; see Fig 1A). These three demonstrators were placed in colonies 6, 7, and 8 (one each; seeded colonies), and bees that came out of the colony were paired up in order of exit from the hive to forage within the arena and tested with the string pulling task. Each bout was capped at 5 min, and we recorded 150 foraging bouts (150 bee pairs). In colonies 9, 10, and 11 (control colonies), no trained demonstrator was present. 150 foraging bouts were recorded (150 bee pairs) (see S2 Data).

Can the combination of multiple simple social learning mechanisms mediate the establishment of a culture-like phenomenon (e.g. group-specific behaviors, such as foraging techniques, that are transmitted via social learning and retained in the group over long periods)? We tracked the diffusion of an experimentally introduced string pulling behavior among foragers of test colonies (Colonies 6, 7, 8) to explore the speed of diffusion and also the retention of the technique in the group beyond the demonstration provided by the first knowledgeable individual. To seed the technique, we trained a single demonstrator per colony to pull the string. Subsequently, we allowed pairs of bees to engage with the string pulling task and tracked the diffusion of the technique among the foraging population (Materials and Methods, Fig 4). Pairs of bees were tested in the order in which they arrived in the corridor connecting the hive to the arena; pairs could be any combination of bees regardless of whether they were naïve, the seeded demonstrator, or a successful learner (S12 Video). As a control, foragers of three separate colonies were tested in the same manner without a seeded demonstrator (Colonies 9, 10, 11).

**Fig 5:**
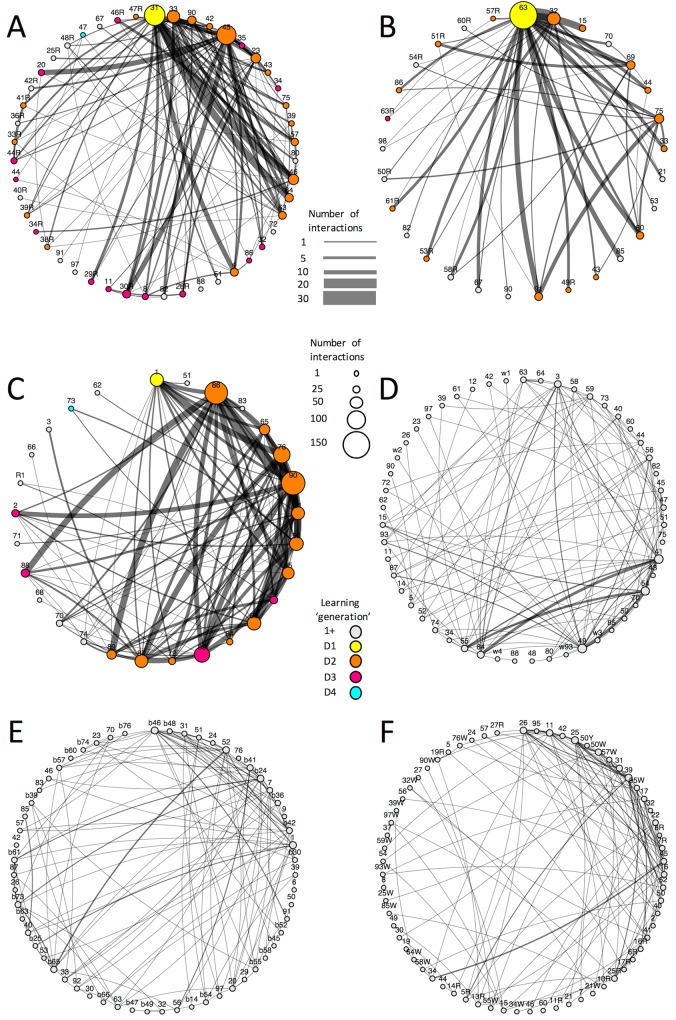
Diffusion of string pulling in bumblebee colonies. (A–F) Nodes represent individual bees. Lines indicate that two bees interacted at least once. Thickness of lines represent total number of interactions between two individuals—one interaction equals one point line thickness and each interaction increases the line thickness by one point. See top insert for indication of line thickness and number of interactions. Size of nodes indicates number of interactions of that individual bee with any other bee—each interaction increases the size of a node by 15% of the original size (3% of the plot width). See middle insert for indication of node size and interactions. Color represents experience (learning “generation”) of that bee: prior to any experience, nodes are grey. After a bee interacts for the first time in the foraging arena, its node turns white. The “seeded” demonstrator (D1), pretrained to pull a string, is marked yellow and at the twelve o’clock position. Once a bee learns to string pull, its node turns from white to another color: orange for a first-order learner (D2, interacting with the seeded demonstrator and lower-order bees); pink for a second-order learner (D3, interacting with first-order and lower-order bees); blue for a third-order learner (D4, interacting with second-order and lower-order bees). See bottom insert for indication of node color and learning generation. Networks for the experiments (A–C) only show interactions within bouts where at least one bee pulled the string at least once. (A) Network for test colony 6 (bout *n* = 189). (B) Network for test colony 7 (bout *n* = 114). (C) Network for test colony 8 (bout *n* = 249). (D) Network for control colony 9 (bout *n* = 149). (E) Network for control colony 10 (bout *n* = 150). (F) Network for control colony 11 (bout *n* = 150) (see S2 Data).

After only 150 paired foraging bouts, a large proportion of each of the test colonies’ forager population (Colony 6: *n* = 25/47, Colony 7: *n* = 17/29, Colony 8: *n* = 12/28) learnt to string pull, whereas none of the control colony foragers (Colony 9, 10, 11: *n* = 51, 58, 57) learnt to pull the string (Fig 5, Materials and Methods, S13–S18 Videos). We conducted additional foraging bouts in two of the tested colonies and found that the technique continued to spread among the foragers for as long as we allowed the spread to progress (Colony 6: 34/47, Colony 8: 18/28, Fig 5, S13 and S15 Videos).

**Fig 6:**
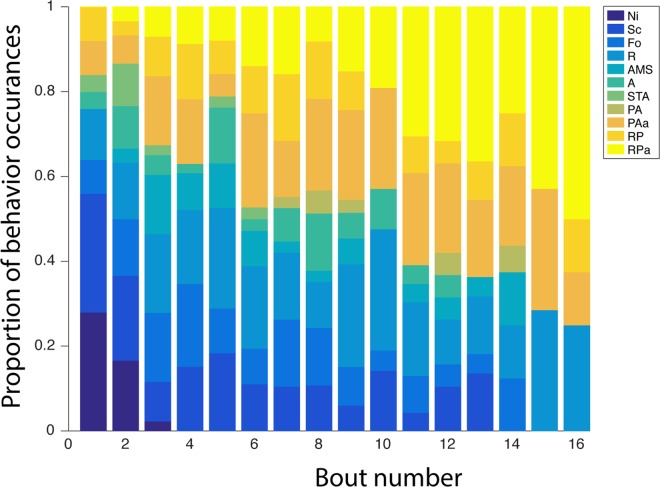
Change in learners’ behavioral interactions. Stacked bars represent the proportion of interactions observed as a function of experience (number of paired foraging bouts). Colors indicate behavioral interactions (abbreviations, see Table 1). We evaluated the behavior of 15 randomly selected individuals (5 from each test colony that had been seeded with a trained demonstrator) for these interactions, scrutinizing 174 5-min videos totaling 14.5 h of footage (see S2 Data).

We quantified the behavioral changes in learner bees over the time of the diffusion experiments. We first screened 81 of the total 419 available videos (~20%) of the paired bouts between demonstrators and learners and inventoried the repertoire of behavioral interactions. We listed 11 types of interactions (Table 1), the frequency of which changed with increasing experience of the learners (Fig 6). Behaviors went through a series of steps with increasing competence, which typically followed the following sequence. During an observer bee’s first few bouts, she would spend most of her time flying around the arena, occasionally landing on top of the table (NI, No Interaction) and spend little or no time near the table, strings, or the other bee. She would gradually start to land beside a bee who had already pulled a string for reward, thereby gaining reward without pulling a string (Sc, scrounging). The observer thus learns to associate the other bee with reward and typically begins following her around the table, keeping in close contact as they both walk (Fo, following). After one or more occurrences of scrounging, the observer bee would begin to reach under the table, sometimes extending her proboscis towards the flower, seemingly in an attempt to gain access to the flower without manipulating the string. While moving around the edge of the table and trying to reach under it, the observer bee might accidentally move a string, but make no subsequent effort to continue moving it (AMS, Accidentally Moving String). Often the observer bee would then position herself next to the bee already pulling a string. She would be in direct contact with the string pulling bee throughout the pull, usually not touching the string (A, Attending), although in some instances ineffectively manipulating the string (STA, String Touching while Attending), and ultimately gaining reward through the other bee’s efforts. Eventually, while in direct contact with a more knowledgeable bee, the observer bee would pull the string, but not enough to move the flower close enough to the edge of the table, extract it, and obtain the reward (PA, Pulling Action with demonstrator). In this phase, she would still rely on the efforts by the more experienced bee to obtain the reward (RP, Rewarded Pull). After more experience, the observer bee would attempt to pull the string on her own without interacting with the other bee, for example, while the demonstrator was flying around the arena. On the first few attempts to string pull on their own, the observer bees did not move the flower enough to be able to obtain the reward (PAa, Pulling Action alone). Finally, after few unrewarded attempts, and typically when paired with a less knowledgeable bee, the observer bee would learn to pull the string on her own to the point of extracting the flower from underneath the table and gaining reward (RPa, Rewarded Pulling alone) and become a trained observer.

**Table 1:** Ethogram of interactions between learners and demonstrators in the diffusion experiment.

Behavioral interaction	Description
No Interaction (NI)	The observer is flying around the arena, is not attracted to the demonstrator, and never lands by the demonstrator or on the table. The demonstrator pulls flowers alone. There is no direct interaction between the two bees.
Scrounging (Sc)	The demonstrator pulls a flower alone. The observer is flying around the arena. Once the demonstrator is drinking from the flower, the observer lands at her side and starts drinking too.
Following (Fo)	The observer is attracted to the demonstrator and follows her for more than 5 s when walking around the edges of the table.
Reaching (R)	The observer tries to reach underneath the table to power her way underneath and sometimes extend the proboscis towards the blue flower.
Accidentally Moving String (AMS)	Whilst walking next to the edge of the table, the observer accidently moves a string. This may move the flower slightly closer to the edge, but the bee makes no further attempt to move the flower via the string.
Attending (A)	The observer is at the side of the demonstrator, in direct contact with her, when she is pulling the string. The observer does not touch or manipulate the string herself. The observer feeds from the flower once the demonstrator finishes pulling the flower from under the table.
String Touching while Attending (STA)	As in Attending (A), but the observer touches the string and tries to manipulate it, however ineffectively (no movement of the flower closer to the edge of the table).
Pulling Action (PA)	The observer pulls the string with her mandibles or legs. The flower moves closer to the edge of the table, though not close enough to allow the bee to obtain the reward. The observer is in direct contact with the demonstrator.
Pulling Action alone (PAa)	Same as above, except that the demonstrator is either flying around or is busy with another flower.
Rewarded Pull (RP)	The observer pulls the string with her mandibles or legs. The flower moves closer to the edge of the table. The other bee is in direct contact with the observer or is trying to pull the same flower. The bees obtain the reward.
Rewarded Pull alone (RPa)	The observer pulls the string with her mandibles or legs. The flower moves closer to the edge of the table. The other bee is either flying around or is busy reaching another flower. The observer (alone) obtains the reward.

These changes in behavior are reflected in the relative frequencies of behavior classes as a function of experience (Fig 6). Whilst nonsocial interactions such as NI and Sc represented more than 55% of the interactions at the onset of the diffusion experiment, they decreased rapidly to 0% over time (Fig 6). In comparison, the percentage of pulling actions displayed by the learners continuously increased with experience from 15% of the interactions at the onset to 60% after 11 bouts. Overall, no major change was observed for the other behavior classes. These results show that learners progressively changed their foraging behaviors from scroungers to competent string pullers.

In test colonies, on average 2 ± 0.06 string pulls were performed per foraging bout and 20 ± 3.9 pulls were displayed per individual over the whole diffusion experiment. Bees needed to be shown 5 ± 0.45 instances of string pulling by an experienced demonstrator before being able to pull the string themselves without demonstration and subsequently demonstrate the technique. Notably, 15 of 104 foragers (Colony 6, 7, 8: *n* = 10, 3, 2, respectively) picked up the technique very rapidly after only one or two observations. There was a significant variation between tested colonies in the average number of string pulls displayed per bee (Colony 6, 7, 8: *n* = 13 ± 4.7, 15.4 ± 9.2, 34.5 ± 7.6, respectively; Kruskal–Wallis test, *H*_*2*_ = 8.790, *p =* 0.012) and the number of observations necessary for a bee to learn the technique (Colony 6, 7, 8: *n* = 4.1 ± 0.4, 7.6 ± 1.1, 5.9 ± 0.9, respectively; Kruskal–Wallis test, *H*_*2*_ = 17.179, *p* ≤ 0.001). In addition, some bees did not manage to acquire the technique despite having been shown the same number of string pulling by other bees (5.6 ± 0.7; Mann–Whitney test, *U*_*93*_ = 1075.5, *p =* 0.261). These results suggest colony and individual variation in social learning ability.

To determine whether experience of the second bee influenced the observer bee’s choice of string to pull, we analyzed the pulling behavior of 25 randomly selected observer bees over the complete sequence of their foraging career during the diffusion experiment (282 paired foraging bouts). We found that observer bees more often pulled the same string as the other bee when paired with a more experienced observer bee or the seeded demonstrator (42 RP instances) than when paired with a less experienced bee (9 RP instances). In contrast, observer bees more often pulled a string alone when paired with a less experienced bee (72 RPa instances) than when paired with a more experienced observer bee or a seeded demonstrator (27 RPa instances).

To test whether bees might cooperate during string pulling, we needed to compare whether experienced bees performed more efficiently when paired with another experienced individual than when foraging alone. Because the diffusion experiment contained only trials with dyads of foragers, the only way to make a direct comparison was to use trials in which an experienced demonstrator was paired with a fully naïve individual that had not shown any pulling action (PA, PAa, RP, or RPa) and thus did not interact or interfere with the skilled forager, who pulled the string singly. Such pairings were compared with instances where both bees were experienced (had already displayed a pulling action). We hypothesized that if cooperation was occurring, strings would be pulled faster and reward obtained quicker in such dyads. However, when paired with an experienced bee, demonstrators (*n* = 16 randomly chosen individuals) took 2.5 times longer to pull the string and obtain the reward (39.9 ± 9 s) than when the same individual demonstrators were paired with an experienced observer who did not interact or interfere with them (15.6 ± 2.1 s; Wilcoxon test, *Z*_30_ = 3.409, *p* < 0.001). These results suggest that bees do not cooperate to pull the string but in fact hinder each other’s efforts to some degree.

Of particular interest for culture-like phenomena is the question of whether a socially learnt behavior routine persists in the population for longer than the original knowledgeable individual can serve as a demonstrator, so that former observers can themselves become demonstrators. If this is the case, then group-specific behavior routines can at least potentially be retained over biological generations. Our network analysis indeed indicates that the technique spread across sequential sets of learners, whereby some bees that learnt the technique never interacted with the seeded demonstrator. In fact, despite the death of the seeded demonstrator in one of the test colonies (Colony 6) after 58 paired foraging bouts, the technique continued to spread among foragers. Moreover we found that there were up to four sequential learning “generations” (as opposed to true biological generations) in two of the three colonies (Fig 5). Learners had string pulling demonstrated to them by up to eight different demonstrators (2.1 ± 0.13), and each demonstrator displayed the technique to 5.3 ± 0.93 learners. Overall, seeded demonstrators displayed eight times more string pulling (119.7 ± 26.5) than the other foragers (14.6 ± 3) (Mann–Whitney, *U*_*68*_ = 4, *p =* 0.004) and demonstrated the technique to five times more foragers (19 ± 2.8) than the other foragers (4.2 ± 0.7) (Mann–Whitney, *U*_*36*_ = 2, *p =* 0.006). This preponderance of the pretrained demonstrators could be a result of higher motivation simply because they obtained reward with every bout, whereas untrained bees often (in the beginning of the experiment) were unrewarded (i.e., unsuccessful until they were paired with a demonstrator or until they learned to pull the string themselves).

To test whether string pulling was diffused socially, we performed network-based diffusion analysis (NBDA). We used the time-based approach described by Hoppitt et al [49]. The Aikake Information Criterion (AIC) was used to determine if string pulling was diffused socially by comparing a social and a nonsocial model for each of the diffusion experiments. We found that for all three experiments, social transmission was more likely than asocial transmission (Table 2).

**Table 2:** Results of network-based diffusion analysis (NBDA). The difference between the fit of the nonsocial model and the fit for the social model is denoted by the change in AIC (ΔAIC). Therefore, positive values indicate a better fit for the social model (p values indicate significance). The social transmission estimate reflects the degree to which social interactions between bees influence the diffusion of string pulling. Positive social transmission estimates that do not cross zero (intervals) indicate significant influence of social interactions.

	ΔAIC	*p*	Social transmission est.	95% CI
*Exp1* (Colony 6)	24.91	<0.000001	9,020.29	952.57–14,392.73
*Exp2* (Colony 7)	5.65	<0.005	2.02	1.13–3.27
*Exp3* (Colony 8)	27.00	<0.000001	6,242.54	3,836.75–9,998.05

We also analyzed the structure of the social networks using exponential-family random graph modeling [50] and found that for all diffusion experiments as well as the control experiments without a demonstrator bee, the structure of the networks was significantly different from random (see Table 3). This indicates that certain bees were more likely to forage together than other bees. Although this could be interpreted as certain individuals preferentially foraging together, given the open-diffusion paradigm and experimental design (in which bees could not freely distribute themselves in space but were forced through the “bottleneck” of the nest entrance tunnel to the foraging arena), this likely reflects temporal factors such differences in when bees began to forage each day, daily changes in foraging activity across bees, and how long each bee takes to return to foraging from the hive.

**Table 3:** Analysis of Social Network Structure for the three experimental and three control colonies. Significant models represent networks where interactions between bees departed from random (i.e., individual bees were more likely to forage with some individuals than others).

	AIC	df	SE	*p*
*Exp1* (Colony 6)	1,559	2,161	0.067	<0.000001
*Exp2* (Colony 7)	646.2	811	0.103	<0.000001
*Exp3* (Colony 8)	917.3	755	0.080	<0.000001
*Ctl1* (Colony 9)	182	1,639	0.251	<0.000001
*Ctl2* (Colony 10)	213.3	2,351	0.237	<0.000001
*Ctl3* (Colony 11)	720.4	2,449	0.112	<0.000001

## Discussion

Here, we show that an invertebrate can be trained to solve a string pulling task, and that a minority can even solve such a task without stepwise training or observation of skilled demonstrators. String pulling is a popular problem-solving paradigm to investigate cognitive abilities in vertebrates [45], in part because scientists in comparative cognition have been interested in exploring the limits of animal intelligence and behavioral flexibility by facing subjects with tasks that are outside their natural repertoires [35,36]. Although there are natural analogues to many standard laboratory tests in animal cognition, string pulling is indeed relatively remote from most animal’s daily behavioral routines. There is no question that many animals regularly pull objects (including bees—e.g., to remove debris or corpses from their nests), but, specifically, the act of object pulling with the purpose of obtaining a food reward, and the learning of such techniques, is not commonly observed in many animals’ daily lives.

As one aspect of exploring animal intelligence, string pulling tasks have been used to test the understanding of means–end relationship: the capacity to mentally model the string as a means to reach an end (the reward) and to understand the connection between the string and the reward. However, most animals appear instead to use perceptual feedback to solve string tasks [45]. Our results indicate that bees may not be different from birds, dogs, or apes in this respect. Bumblebees relied on the perceptual feedback provided by their actions, resulting in target movement to learn string pulling, and failed, for example, in an experiment in which the string was coiled, and therefore tugging on it did not result in immediate feedback. However, through experience, bees eventually learnt to associate the string with the reward and solved the task without the need for feedback. Nonetheless, this would not allow bees to solve tasks requiring means–end understanding such as the discrimination of connected and disconnected strings.

More than a century of research in social learning in animals has revealed a plethora of evidence that animals, from primates and cetaceans to birds and fish, can acquire novel skills by observing the actions of others [1,3,5,6,30,51]. Growing evidence also shows that insects can glean critical information about their environment by observing others [52–54]. Here, we show that uninformed bumblebees can learn a novel and highly unnatural foraging technique, string pulling, by observation. To this end, our bees used a combination of simple forms of learning. Consistent with a previous study [55], during observation, bees were able to pick up the location of a new access to the reward (local enhancement of flower position). In addition, we showed that the observers were attracted to the string (stimulus enhancement). A recent study showed that learning about rewarding flowers from conspecifics resulted in stimulus enhancement, whilst learning from nonsocial or model demonstrators resulted in local enhancement [46]. In contrast, our results suggest that observers can use both forms of information independently of the cue type. In addition, the results of the coiled-string experiment also indicate that trial-and-error learning was involved in learning the technique. That is, when observing the demonstrations, the observers did not learn the specific sequence of actions used by the demonstrator with the string but simply knew to go to the correct location where the string was accessible. They had to learn the technique of how to move the target (blue flower) closer by individual exploration. These results suggest that the combination of relatively simple forms of social learning and trial-and-error learning can mediate the social learning of novel skills [19,29,56].

In this sense, our study adds to the growing evidence that simple principles of “asocial” associative learning can also account for many aspects of social learning [29,53]. For instance, observational learning about flower colors in bumblebees can emerge through the simple Pavlovian ability to integrate two learned associations (second-order conditioning) [28]. This mechanism has more commonly been explored in nonsocial learning and is also common to social and solitary species [28,57]. Moreover, social learning ability and asocial learning ability covary across and within species [57]. Overall, this suggests that social and asocial learning are mediated by the same “generic” mechanism [29,57]. The use of generic mechanisms in learning generates the possibility to combine different forms of learning, allowing bees to use local as well as stimulus enhancement and trial-and-error learning to learn string pulling by observation.

Even if social and asocial learning rely on common associative learning mechanisms, the sensory filters that allow animals to recognize conspecifics can guide attention of observers to valuable resources [58]. This may explain why observer bees were not able to solve the task in the “ghost experiment.” Without the presence of a visible demonstrator, the motivation or the attention paid by the observer to the flower movement and the string may not have been sufficient to learn the critical information required to solve the string task [57], or, indeed, bees may not have paid attention to the action of the moving flower. Consistent with this, a recent report on learning in bees suggested that the specific attention directed to mobile salient cues provided by conspecifics could explain the dissociation of social and asocial learning [53].

The spread of novel foraging techniques has often been viewed as evidence that animals have the basic cognitive tools needed for cultural transmission of skills [34,41,59]. From an evolutionary perspective, culture should allow for the passing along of advantageous information through generations of learners. Here, we show that a novel, experimentally seeded foraging technique can spread through social learning by observation in bee populations. Moreover, we report that the novel routine persisted in the population for longer than the original knowledgeable individual served as a demonstrator, so that successive learning “generations” became demonstrators. Though these sequential sets of learners are not true biological generations, these results indicate that, just as in birds [60] and mammals [61], an experimentally introduced innovative behavior can spread via cultural transmission in social insect groups and potentially be retained over long periods. Together with recent work documenting social learning in fruit flies [62], our results suggest that insects possess the essential cognitive elements for cultural transmission.

It may be asked what the natural relevance of our findings is, or whether there is likely to be a natural analogue of the cultural transmission of a unique foraging routine as we have described in bumblebees. This may be unlikely to be the case, but our results indicate that this is a question of opportunity rather than a question of whether or not bumblebees have the cognitive toolkit to exhibit culture-like processes. We found that when the appropriate social and ecological conditions are present, culture can be mediated by the use of a combination of simple forms of learning [28,63]. Thus, cultural transmission does not require the high cognitive sophistication specific to humans, nor is it a distinctive feature of humans.

It may well be that the absence of such cultural transmission phenomena in bees and other animals in the wild simply reflects the absence of natural opportunities. For example, the spread of the milk bottle opening “culture” in some British songbirds in the 20th century only arose because humans created a “niche” for the behavioral spread of this technique to become useful (by depositing accessible milk bottles on doorsteps of households hours before they were collected) [6]. If a majority of United Kingdom households presented artificial flowers that require unusual manipulation techniques to pollinators, especially at times of dearth, it is equally conceivable that these manipulation techniques would spread socially through pollinator populations. If nature presented such challenges, and if bee foraging activities were not discontinued during winter months, it is clear from our work that bees have the learning capacities to affect long-term, group-specific behavior patterns.

More sophisticated forms of social learning and cognitive mechanisms specific to human culture may well have evolved from simpler forms of learning and cognition as described here.

Human culture exhibits unparalleled complexity and diversity, and is unambiguously cumulative in character [21,64]. The combination of high-fidelity transmission (e.g., via imitation, teaching, language) of beneficial modifications of cultural knowledge with the ability to identify “who knows” these modifications with greater accuracy and precision by metacognitive representation promotes cumulative culture in humans [63]. Despite the obvious differences between humans and other animals, understanding social learning and culture in animals holds a key to understanding the evolutionary roots of the peculiarities of social learning and culture in humans. It is clear from our study and others on cultural diffusion in animals that once experimenters create the conditions under which such diffusion is beneficial (often via allowing access to desirable nutrition via man-made devices that must be operated in specific ways), they can be instantly observed in many animals. Early tool-using hominids are likely to have created the conditions for themselves that favored the further evolutionary fine-tuning of social learning processes that results in high-fidelity transmission and cumulative culture [21,64]. Our findings add to the accumulating evidence suggesting that the capacity of culture may be within most animals with a relatively basic toolkit of learning processes as described here, in turn shedding light on the evolutionary precursors of the more sophisticated forms of culture in humans.

## Materials and Methods

### General Methods and Animal Model

*Bombus terrestris* foragers from 11 colonies obtained from a continuous rearing program (Biobest, Belgium N.V.) were used for the experiments. Bumblebee nests were kept in 40 × 28 × 11 cm bipartite wooden nest boxes. Colonies were provided with 7 g commercial pollen (Koppert B.V., The Netherlands) every 2 d. Through a Plexiglas corridor (25 cm length, 3.5 × 3.5 cm in cross-section), bees were allowed access to a flight arena (100 × 75 × 30 [height] cm) where they were trained and tested. Three plastic sliding doors located along the corridor allowed controlled access to the arena. Before training and tests, all the bees were pretrained to associate blue artificial flowers (3 cm diameter blue discs with an inverted Eppendorf cap at the center) with the reward (30% sucrose solution, w/w). Pretraining consisted of bees foraging freely for 1 h on a patch of six blue artificial flowers (ad libitum reward, Step 0, Fig 1A) randomly located in the arena. This phase allowed for the experimenters to identify regular foragers that could be used in individual training. Training and tests were conducted between 9 a.m. and 7 p.m. under standardized light (12:12, high-frequency fluorescent lighting [(TMS 24F) lamp with HF-B 236 TLD (4.3 Khz) ballasts, Phillips, Netherlands, fitted with Activa daylight fluorescent tubes, Osram]) and temperature (25 ± 2°C) conditions at the Bee Behavioural and Sensory Ecology Laboratory (Queen Mary University of London). Five colonies (1–5) were used, two for the string pulling acquisition experiment (Colonies 1 and 2), two for the social learning experiment (Colonies 1 and 3), and three to explore the mechanisms of social learning in string pulling (Colonies 1, 4, and 5). In these experiments, bees were allocated randomly to the demonstrator, observer, or untrained group, and individuals were never used in different groups. We trained bees individually to string pull on one day and then used the trained individuals to demonstrate the technique to observers on subsequent days. Six different colonies were used for the cultural diffusion experiment (Colonies 6–11). In this experiment, we chose to train the bee that seemed to forage with regularity to seed string pulling in tested colonies. The other foragers were by default observers and became demonstrators once they learnt the technique. At the end of a training or testing day, bees were again allowed to freely enter the arena to forage from six blue, openly accessible artificial flowers (ad libitum reward) for 1 h. After testing was complete, tested bees were freeze-killed. To examine whether size influenced success, measurement of the bee thorax width were taken with an electronic digital caliper (NewOctave Global, Astoria, United States, precision of ±0.02 mm).

### The Acquisition of a String Pulling Technique by Individual Bumblebees (Colonies 1–11)

#### Tests with Untrained (Naïve) Individuals (Colonies 1–11)

To test the capacity of bees to “innovate” string pulling, naïve individuals (*n* = 291) were challenged with a string task (Test 1). Every bee used in this study went through Test 1 after the pretraining association between the flower and the reward. Bees were individually tested in the arena and presented with three blue artificial flowers with a string (three twisted cotton threads, length = 4.5 cm, 0.3 cm of diameter) glued (superglue) to each and placed under a small transparent Plexiglas table (18 × 20 × 0.4 cm, S1–S4 Videos). Artificial flowers were each rewarded with 50 μl of 30% sucrose solution. The table was 0.6 cm above the ground so that bees could not squeeze underneath to reach the sucrose solution. Individuals were given 5 min to solve the task and then were returned to the colony. Twenty-five bees (Colony 1) were given a second 5-min opportunity to solve the string task.

#### String Pulling Training (Colony 1 and demonstrators in all other experiments)

During training on the string pulling task, selected bees (*n* = 40, Colony 1) were challenged to obtain the reward when the flowers were gradually positioned further under the table (Steps 1–4, Fig 1A). The arena was set up the same as for Test 1. Only one bee was allowed into the arena at one time. To obtain the reward in Step 1, bees were required to locate the flower partially under the Plexiglas table. In this scenario, bees could gain access to sucrose solution without moving the flower. Steps 2 and 3 required the bees to move the flower (75% and 100% covered) in order to access the reward. In the final step (4), bees had to use the string to pull the flower from 2 cm under the table (Fig 1A). Most of the time, bees exposed just enough of the flower to get the reward (approximately half). However, once the flower was empty of reward, bees often pulled the entire flower from underneath the table, possibly expecting more reward. For Steps 1–3, bees (*n* = 40, 32, and 29, respectively, Colony 1) were given five foraging bouts of 5 min each to learn to solve the tasks. In the fourth step, the reward was accessible only by pulling the string. Strings protruded from the table by 2.5 cm. Bees (*n* = 28) were tested ten times after the first occurrence of string pulling during the fourth step. Bees that did not succeed in obtaining the reward during any of the ten tests in Step 4 were not used as demonstrators later in the study. Bees that stopped foraging for more than a day also were not used as demonstrators.

#### Influence of Perceptual Feedback on String Pulling (Colony 2)

We assessed the influence of perceptual feedback and experience on string pulling by challenging 15 successfully trained bumblebees (see String Pulling Training) with a string pulling task in absence of salient colored stimuli. We removed the blue disc from the artificial flowers and attached strings to the transparent Eppendorf inverted cap containing the reward. We placed three colorless flowers under the Plexiglas table and challenged individual bees to obtain the reward following the procedure of training Step 4. Bees were tested a first time immediately after training on the same day. Then they were tested a second time after having performed 20 instances of string pulling over 48 h (10 instances per 24 h).

### Social Learning of String Pulling by Caged Observers (Colonies 1 and 3)

#### Social Observation (Colony 1)

We selected bees that were trained to forage on blue artificial flowers but had no prior experience with strings to become observers. Selected individual naïve bees (*n* = 25) were placed manually into a Plexiglas chamber (5.5 × 3.6 × 3.6 cm). The chamber was opaque on the front, back, and top, leaving only the left and right sides transparent. We initially experimented with a transparent top lid of the observation chamber, because we planned to monitor observer behavior in the presence of demonstrators. However, in this setting, observer bees were positively phototactic and spent a large percentage of the time trying to escape the chamber at the top. Adding a nontransparent lid to the observation chamber narrowed the bees’ view to the tables and flowers (and demonstrator). Hence it was not possible to videotape the observers’ behavior or their gaze direction in the observation chamber. We trapped the selected individual in between two sliding doors and positioned the transparent chamber at the end of the tunnel. Then we let the bee walk into the transparent container, locked it with a piece of tape, and positioned the chamber with the observer in between two small transparent tables (8 × 8 cm, 0.6 cm above the ground). The center of the chamber was equidistant (8 cm) to the center of the two tables (Fig 2A). At this distance, the diameter of the string would have subtended 3° and therefore would have been detectable by bees in the observation chamber [65], as also evidenced by the behavior results when the string was presented at unexpected locations (see Results section). Bees were given 2 min to acclimatize to the chamber and were provided with a tiny drop of sucrose solution (5 μl) in the center of the chamber. Then, bees were subjected to the observation phase: one experienced demonstrator bee of the same colony was released in the arena and allowed to gain access to the reward by pulling the string (Fig 2B). Each foraging bout (duration: 2–5 min) involved one individual demonstrator, but the whole sequence of five bouts was completed by one or two different trained foragers. Because flowers provided 50 μL reward (about half of a bee’s stomach capacity), demonstrators had to collect food from two artificial flowers to fill their crop, i.e., displayed the string pulling technique twice for each foraging bout on both sides of the chamber. Once their crop was filled, demonstrators flew back to the tunnel entrance to return to the colony. After each demonstrator foraging bout, the flowers were manually refilled and repositioned. Each observer had the opportunity to observe ten instances of string pulling, five times on each side of the chamber. After the observation phase, the tested bee was released in the arena and given 5 min to solve the string pulling task (Test 2, Fig 2B). To prevent the use of chemosensory cues, in this experiment and all others, new strings were attached to flowers and the arena ground, and the transparent tables were washed with hot water and ethanol before testing the observers. Trials were videotaped with a Sony camcorder (Handycam HDR-CX 190E, Sony, Tokyo, Japan) placed above the arena to analyze the bees’ behavior, times taken to obtain the reward, and the locations they explored.

#### “Ghost Control” (Colony 3)

To test whether observers (*n* = 15) could learn to string pull without demonstration of the handling procedure, we repeated the social observation paradigm, except that two 30 cm nylon threads (transparent, 0.5 mm diameter) were attached to the strings of the two artificial flowers [40]. Two holes were drilled in the arena wall on both sides of the entrance tunnel to let the nylon threads protrude outside the arena. At a distance of 6.5 cm, the diameter of the nylon thread would have subtended just 0.4° and therefore would not have been detectable by bees in the observation chamber [65]. Naïve observers were again locked in the observation chamber, as described in the previous section. However, in this case the string pulling was not performed by a demonstrator bee, but by the experimenter via the nylon thread. During the observation phase, the experimenter pulled one of the threads every 3 min and immediately let one forager (trained to visit blue flowers, but untrained with the string pulling task) into the arena. It is important to note that bees could not have observed human fingers pulling the string, because the string was pulled from outside the arena. Once the forager had emptied the first flower, the experimenter pulled the second flower to allow the forager to fill its crop and return to the colony. After ten demonstrations, the observer was released in the arena and challenged with the string task (Test 2, Fig 2B).

### The Mechanisms of Social Learning in String Pulling (Colonies 1, 4, and 5)

#### Local Enhancement (Colony 1)

To examine the possibility that bees were using local enhancement to solve the string pulling task, we video analyzed the behavior of bees of the social learning experiment. We delineated four regions—the area in front of the table where the string was located during observations, the side opposite the string, the adjacent sides, and the area on top of the table (Fig 3A)—and we assessed whether observers and untrained bees spent more time in the zone where the demonstrator was present during the observation phase. Because successful observers spent most of their time in the region with the string (because they spent most of the time pulling the string to obtain the reward), we only compared the behavior of observers that were not able to solve the task (*n* = 10) to untrained bees (*n* = 23) to show that even if they were unsuccessful, the observation had an effect on their behavior, i.e., they used the visually obtained social information available to some extent.

#### Stimulus Enhancement (Colony 4)

We tested whether observers (from Colony 4, *n* = 14) were attracted to the presence of the string. We repeated the procedure of the social learning experiment, with the modification that during the test phase, the flowers were placed so that the string protruded from the opposite side on one side of the tables and at 90° to the training location on the other (Fig 3A). Behavior was videotaped with a camera (Handycam HDR-CX 190E, Sony, Tokyo, Japan) placed above the arena and the recordings were analyzed following the methods described in the test of local enhancement.

#### Perceptual Feedback and Trial-and-Error Learning (Colony 5)

Bees (*n* = 27) were tested with a coiled-string paradigm after having observed demonstrators pulling straight strings to obtain reward ten times (methods identical to the social learning experiment). After the observation phase, however, we replaced the straight strings with 14-cm-long strings that we coiled in a zigzag pattern (four line segments) under the tables so that only 0.5 cm of the string protruded (Fig 2B). Once released, the observer was given 5 min to solve the task.

### The Spread of String Pulling in a Transmission Chain Experiment (Colonies 6–11)

We tested bees in pairs in an arena set up with four artificial flowers with strings (Fig 4). Colonies 6–8 were each seeded with a single demonstrator, whereas colonies 9–11 only included untrained foragers. This is an “open diffusion” design [5], insofar as forager pairings were left open and not constrained by the experimenter. Such open diffusion experiments more closely simulate natural foraging conditions than alternatives such as highly constrained linear transmission chain designs [39]. We briefly explored a fully “open” design that allowed unlimited foragers into the flight arena, but this resulted in a “frenzy” of multiple foragers piling on top of each other near the artificial flowers, and this did not allow us to monitor which individuals learnt from which demonstrators. Therefore, the only constraint upon the openness of the diffusion was that we limited the maximum number of individuals that entered the arena to two, on a first-come, first-serve basis: the first two individuals that entered the tunnel leading to nest, irrespective of these foragers’ identities and prior information, were allowed into the experimental arena. Upon release, the pair of bees was given 5 min to solve the task. We videotaped each foraging bout and recorded whether individuals pulled the strings and drank from the flowers. We tested 150 paired foraging bouts per colony (Colonies 6–11) and tracked the diffusion of string pulling behavior among the foragers. In two of the tested colonies (Colonies 6 and 8), we conducted 95 and 39 additional foraging bouts to assess whether the technique would continue to spread. We mapped the diffusion of the technique on a social network created using a customized version of the R package ggnetwork (version 3.2.2, Fig 5). We conducted a second-by-second video analysis of 81 bouts (four randomly selected bees per test colony) to inventory the behavioral interactions between learners and demonstrators and make the ethogram. Table 4 summarizes all treatments, sample sizes, and success rates.

**Table 4:** Experiment Summary Table. Summary of all experiments, including name, number of colony or colonies used, sample size, and success rate of observed individuals.

Experiment	Colony	Success rate N
String Pulling Training	1	23/40
Solution of String Pulling by Untrained Bees (Test 1)	1–11	0/291
Solution of String Pulling by Untrained Bees (Test 2)	1, 9–11	2/135
Perceptual feedback in demonstrators with little experience	2	2/15
Perceptual feedback in demonstrators with extensive experience	2	11/15
Social Observation	1	15/25
“Ghost Control”	3	0/15
Stimulus Enhancement	4	0/14
Coiled-String Experiment in Observers	5	0/27
Coiled-String Experiment in Trained Demonstrators	5	3/8
Transmission Chain Experiment (with seeded demonstrator)	6	25/47
	7	17/29
	8	12/28
Transmission Chain, Control (without seeded demonstrator)	9	0/51
	10	0/58
	11	0/57

## Supporting Information

S1 Data Excel spreadsheet containing data plotted in Figs 1–3 and the data supporting the results (text) for the body size influence on string pulling, the colorless flower experiment, the “ghost experiment,” the coiled-string experiment, and the observation during demonstration. (XLSX)

S2 Data Excel spreadsheet containing the raw data of Colonies 6–11 used in the transmission chain experiment and supporting the network analysis illustrated in Fig 5, the data values plotted in Fig 6, and the data supporting the test of the influence of the observer bee’s choice and cooperation. (XLSX)

S1 Video String pulling training Step 1. Footage shows a bumblebee in training Step 1, obtaining sucrose solution from an artificial blue flower disk partially (50%) covered by the transparent table. The bee locates the blue flowers, lands in the center of the middle flower at the edge of the table, and drinks from the inverted Eppendorf cap.
(MP4)

S2 Video String pulling training Step 2. Footage shows a bumblebee in training Step 2 obtaining sucrose solution from a flower that is three-quarters covered by the transparent table. The bee lands at the edge of the table and immediately repositions herself in front of the table. She then steps onto the blue flower and moves her forelegs and middle legs back and forth while pushing against the edge of the table with her head to slide the flower out. Simultaneously, she extends her proboscis between the flower and the table. As soon as the proboscis comes into contact with the sucrose solution, the bee starts drinking and stops moving her legs.
(MP4)

S3 Video String pulling training Step 3. Footage shows a bumblebee in training Step 3 obtaining sucrose solution from a fully covered flower positioned at the edge of the table. The bee lands on top of the table and immediately repositions herself in front of the table. Then, she starts extending her proboscis in between the flower and the table whilst manipulating the string with her middle and forelegs, grasps the edge of the flower with her forelegs, and pulls it with both legs alternately. Once the flower is extracted, she steps onto it, moves her forelegs and middle legs back and forth while pushing with her head against the edge of the table to slide the flower out, and obtains the reward as in Step 2.
(MP4)

S4 Video String pulling by an experienced bumblebee. Footage shows an experienced bumblebee worker pulling a string to extract the artificial blue flower disk from underneath the transparent table and subsequently drinking sucrose solution found in the center of the flower (Step 4). The bee lands in front of a string, grasps it with her forelegs, and pulls the string with both legs alternately. She also uses her mandibles and extracts the flower from under the table by moving her head upward and backward. Finally, the bee steps onto the blue flower and moves her forelegs and middle legs back and forth while pushing with her head against the edge of the table to slide the flower out from underneath the table to access the reward.
(MP4)

S5 Video String pulling without colored stimulus. Footage shows an experienced bumblebee demonstrator worker solving the string task when the blue flower disk has been removed. Released into the arena, the bee flies close to the string several times, but she does not land in front of or on top of the table for 4 min. The bee even tries to go back to the colony a few times. Eventually she lands in front of the left table, pulls the string, and obtains the reward using the technique described in Video S4.
(MP4)

S6 Video Successful string pulling by an untrained bumblebee, or “innovator.” Footage shows one of the two untrained bumblebee workers that ever managed to solve the string task without stepwise training or observation of skilled demonstrators. This bee was exceptionally explorative and tried a wide variety of methods. She initially lands on top of the table several times and tries to obtain the reward. After 2 min and 30 s, the bee lands at the edge of the table above the left side flower and tries to obtain the reward in the inverted position. For a few times, the bee walks off the table, tries to push her way underneath, extends her proboscis toward one of the flowers, and walks back on top of the table. After 3 min, whilst trying to push her way under the table towards the left flower, the bee accidentally grasps the string with her left middle leg and, moving her leg back and forth, pulls the string. The flower moves closer and the bee extends her proboscis between the flower and the table, but the reward is still out of reach. The bee keeps pulling the string with her left middle leg, moving the flower closer, but simultaneously pushes the flower back under the table with her extended proboscis. Then, she repositions herself on the other side of the string, grasps, and pulls the string by moving her right middle leg back and forth. As a result, the flower moves to the edge of the table. Using the same movement of the same right leg, the bee grasps the blue disk and extracts the whole flower from underneath the table. Finally, she steps onto the flower and walks to the center to obtain the sucrose.
(MP4)

S7 Video String pulling after observation of a skilled demonstrator by a previously naïve forager. Footage shows an observer bumblebee pulling a string to drink sucrose solution from the flower placed underneath the transparent table immediately after the observation phase. The observation chamber is positioned between two transparent tables with flowers and strings. At first, the observer bee lands at the edge of the right table and tries to reach under the table in an inverted position, with the body curved around the edge of the table, the back legs on top of the table, and the middle and forelegs underneath the table. The observer extends her proboscis toward the flower, moves the string from side to side with her fore and middle legs, and walks away. She then lands few times on top of the left and right table and tries to access the reward. After 2 min, the observer lands at the edge of the left table, repositions herself in front of the string, and starts moving the string sideway with her forelegs. Following this, the observer grasps the string with all of her legs and pulls it for the first time. The flower moves closer but not enough to obtain the reward. The bee then releases the string, steps onto the table, and comes back to the string. Moving both her fore and middle legs alternately, the bee grasps the string, pulls it a second time, and extracts the flower from underneath the table. Finally, she steps onto the blue disk and slides the flower to obtain the reward using the same technique as in S4 Video.
(MP4)

S8 Video “Ghost” experiment. Footage shows a nonsocial observer bumblebee worker tested with the string task after the observation phase. The bee lands several times on top of the left and right tables and tries to obtain the sucrose. In getting off the table, the bee accidently moved the string sideways but did not appear pay attention to this movement. After 4 min, the bee eventually lands at the edge of the left table and tries to reach the flower by pushing her way under the table. The bee touches the string with her fore and middle legs but does not grasp or pull it. Twice, the string moves sideways because the bee walked on it, with no consequence on the flower position. The bee keeps coming back to top of the table and remains ultimately unsuccessful.
(MP4)

S9 Video Test of stimulus enhancement. Footage shows an observer bumblebee tested with the string task after the observation phase. Whilst the strings protruded in the normal region during in the observation, they protruded in two alternative regions during the test. For a few times, the bee lands on top of the left and right tables and tries to obtain the sucrose solution. After 50 s, the bee lands on the opposite side of the right table, tries to reach under the table, moves the string with her middle legs, moves the flower a little closer, and flies away. Then the bee lands several times on the table tops. After 3 min and 40 s, she lands on the left table, walks to the edge where the string protrudes, and subsequently leaves the table. Then, she manipulates and grasps the string with all her legs, gets her body around the string in an inverted position, and pulls the flower a little closer to the edge. Finally, she stays in an inverted position with her body under the string, grasping the string with all her legs, and extends her proboscis towards the edge and under the table.
(MP4)

S10 Video Coiled-string experiment (observer). Footage shows a bumblebee attempting to solve the coiled-string task after having observed a skilled demonstrator. The bee lands on top of the right and left tables several times and tries to access the reward. After 1 min and 40 s, the bee gets off the left table and starts exploring one of the edges for 5 s. The bee then climbs back onto the table, flies away, and attempts to obtain the reward from the top several more times. After 4 min, the bee lands on the right table, gets off it, and comes in contact with the string. She tries to power her way under the table where the string protrudes, walks around the table, and explores different edges of the two tables. She eventually climbs back on top of the left table and walks away. After 4 min and 30 s, the bee lands again on top of the right table, gets off it, and touches the string. She then tries to push her way under the table and doing so accidently moves the string with her legs. The flower doesn’t move closer and the bee keeps trying to push her way under the table and tries this on different edges. She finally climbs back onto the table, walks straight to the string side, and tries to reach under the table in an inverted position, extending the proboscis, ultimately failing to reach the reward.
(MP4)

S11 Video Coiled-string experiment (demonstrator). Footage shows an experienced bumblebee solving the coiled-string task. The bee first lands on top of the tables and tries to access the reward, gets off the left table, positions herself at the edge of the table, extends the proboscis, and starts manipulating the string. Using her fore and middle leg, the bee grasps and pulls the string to the second bend. Next, the bee clenches the second bend with her mandibles and walk backwards to pull the string. Then the bee grasps the string with her fore and middle legs and, moving them back and forth, pulls the string, following the zigzag, and extracts the blue flower. Finally, she steps onto the flower and slides the flower out and accesses the reward using the technique described in S4 Video.
(MP4)

S12 Video Open diffusion experiment. Footage shows a pair of bees (the seeded demonstrator and an observer) tested with the string pulling task in Colony 8. The red dot indicates the seeded demonstrator. The observer has not learned string pulling yet but has already been tested three times in paired foraging bouts. The demonstrator lands at the edge of the table, repositions herself in front of the string, and starts pulling immediately. The observer is first attracted to the blue flower and lands on top of the table. The observer subsequently flies to the demonstrator, lands at her side, and walks to the nearby flower and string. She walks along the protruding string, reaches the table edge, and moves sideways. She notices the demonstrator and walks to her side, moving around her whilst the demonstrator is pulling, always in close contact. The observer touches the string a few times but does not grasp it. The demonstrator eventually extracts the blue disk and steps onto it. The observer copies the demonstrator. They both slide the flower from under the table and obtain the reward. Once the first pulled flower is depleted, the demonstrator moves to the nearest flower and pulls the string. The observer stays on the extracted flower for a short period, circling, probing the emptied inverted cap before noticing the demonstrator drinking from a second flower and joining her. In a similar way, once the second pulled flower is emptied, the demonstrator moves and pulls a third flower and the observer joins her. Her crop filled up, the demonstrator flies back to the colony.
(MP4)

S13 Video Diffusion of string pulling behavior through the social network of Colony 6 (bout *n* = 189). Nodes represent individual bees. Lines indicate that two bees interacted at least once. Thickness of lines represent total number of interactions between two individuals—one interaction equals one point line thickness, and each interaction increases the line thickness by one point. Size of nodes indicates number of interactions of that individual bee with any other bee—each interaction increases the size of a node by 15% of the original size (3% of the plot width). Color represents experience (learning “generation”) of that bee: prior to any experience nodes are grey. After a bee interacts with another bee for the first time in the foraging arena, its node turns white. The “seeded” demonstrator, pretrained to string pull is yellow and at the twelve o’clock position. Once a bee learns to pull a string, its node turns from white to another color: orange for a first-order learner (interacting with the seeded demonstrator or lower order bees); pink for a second-order learner (interacting with first-order or lower-order bees); and blue for a third-order learner (interacting with second-order or lower-order bees).
(MP4)

S14 Video Diffusion of string pulling behavior through the social network of Colony 7 (bout *n* = 114). For further explanation, see S13 Video legend.
(MP4)

S15 Video Diffusion of string pulling behavior through the social network of Colony 8 (bout *n* = 249). For further explanation, see S13 Video legend.
(MP4)

S16 Video No spread of string pulling in the absence of a seeded demonstrator in the network of control Colony 9 (bout *n* = 149). For further explanation, see S13 Video legend.
(MP4)

S17 Video No spread of string pulling in the absence of a seeded demonstrator in the network of control Colony 10 (bout *n* = 150). For further explanation, see S13 Video legend.
(MP4)

S18 Video No spread of string pulling in the absence of a seeded demonstrator in the network of control Colony 11 (bout *n* = 150). For further explanation, see S13 Video legend.
(MP4)

## Abbreviations

A: Attending
AIC: Aikake Information Criterion
AMS: Accidentally Moving String
Fo: following
NBDA: network-based diffusion analysis
NI: No Interaction
PA: Pulling Action with demonstrator
PAa: Pulling Action alone
R: reaching
RP: Rewarded Pull
RPa: reward pulling alone
Sc: scrounging
STA: String Touching while Attending
