# Correction: Associative Mechanisms Allow for Social Learning and Cultural Transmission of String Pulling in an Insect

**DOI:** 10.1371/journal.pbio.1002589

**Published:** 2016-12-29

**Authors:** Sylvain Alem, Clint J. Perry, Xingfu Zhu, Olli J. Loukola, Thomas Ingraham, Eirik Søvik, Lars Chittka

In Fig 2C, both Test 2 bars for Untrained and Observer (social) bees are slightly higher than they should be. Although the number inserts within the bars are correct (2/25 and 15/25) and the information within the manuscript text is reported accurately, the bars themselves are incorrect and should be at heights indicating 8% and 60%, respectively, as reported in the main text. There are no changes to the figure legend and the corrected figure has been provided here.

**Fig 2:**
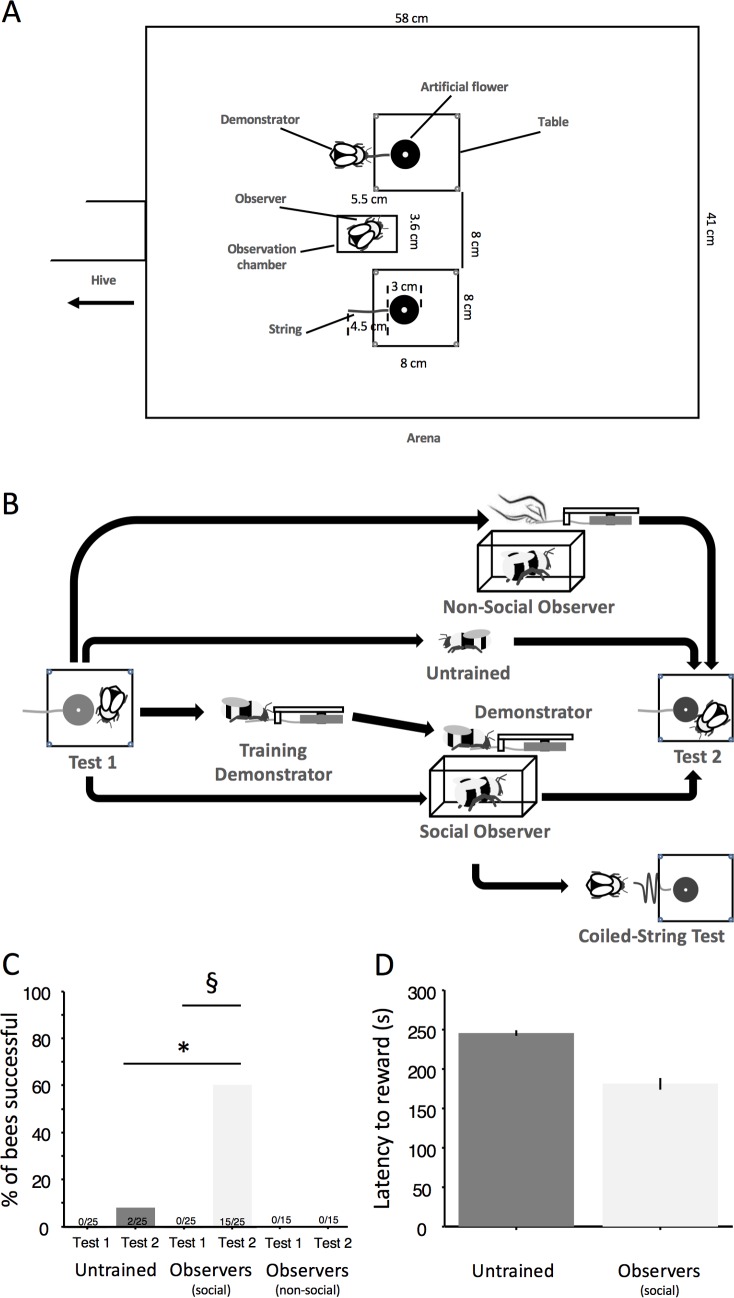
Social transmission of string pulling. (A) Arena set up for the observation of string pulling. (B) The various testing procedures. Tests 1 and 2 were identical and consisted of giving 5 min to individual bees to solve the string pulling task. After having been trained to forage from blue artificial flowers, bees were tested a first time (Test 1). Then, demonstrators were trained (see Fig 1) and used to display string pulling (two instances, straight strings) during each of five foraging bouts to individual observers (*n* = 52) placed in a transparent Plexiglas cage. After the observation phase, 25 observers were tested again with the straight-string task (Test 2) and 27 with the coiled-string task. Fifteen different bees observed the flower moving without visible actor so that a forager could then obtain the sucrose solution (“Ghost control”) and, where tested, with the straight-string task subsequently. Untrained bees (*n* = 25) were also tested a second time with string pulling. (C) Percentage of successful untrained, social, and nonsocial observer bees in Tests 1 and 2. Asterisk: Fisher’s exact test, *p* ≤ 0.0001. Double S: McNemar test, χ^2^_1_ = 13.067, *p* < 0.001. (D) Mean ± s.e. (s) latency in accessing the reward in untrained and observer bees. Observers’ latency was not different from that of the two “innovators” (Mann–Whitney *U* test, U_15_ = 6, *p* = 0.205), (see S1 Data).

Secondly, we report an incorrect value (N = 57) for the control colony #11 in Table 4 of the manuscript file and in the Results section, under the heading “The Spread of String Pulling in a Transmission Chain Experiment” (page 10 of the PDF). Fig 5 shows the correct number of nodes indicating the colony size (N = 66) and all related data analyses use the correct value.

**Table 4:** Experiment Summary Table. Summary of all experiments, including name, number of colony or colonies used, sample size, and success rate of observed individuals.

Experiment	Colony	Success rate N
String-Pulling Training	1	23/40
Solution of String Pulling by Untrained Bees (Test 1)	1–11	0/291
Solution of String Pulling by Untrained Bees (Test 2)	1, 9–11	2/135
Perceptual feedback in demonstrators with little experience	2	2/15
Perceptual feedback in demonstrators with extensive experience	2	11/15
Social Observation	1	15/25
“Ghost Control”	3	0/15
Stimulus Enhancement	4	0/14
Coiled-String Experiment in Observers	5	0/27
Coiled-String Experiment in Trained Demonstrators	5	3/8
Transmission Chain Experiment (with seeded demonstrator)	6	25/47
	7	17/29
	8	12/28
Transmission Chain, Control (without seeded demonstrator)	9	0/51
	10	0/58
	11	0/66

The corrected text and Table 4 can be found here.

“After only 150 paired foraging bouts, a large proportion of each of the test colonies’ forager population (Colony 6: n = 25/47, Colony 7: n = 17/29, Colony 8: n = 12/28) learnt to string pull, whereas none of the control colony foragers (Colony 9, 10, 11: n = 51, 58, 66) learnt to pull the string (Fig 5, Materials and Methods, S13–S18 Videos).”

These corrections do not change the results or conclusions of the paper.
